# Detection of *trans*–*cis* flips and peptide-plane flips in protein structures

**DOI:** 10.1107/S1399004715008263

**Published:** 2015-07-28

**Authors:** Wouter G. Touw, Robbie P. Joosten, Gert Vriend

**Affiliations:** a Centre for Molecular and Biomolecular Informatics, Radboud University Medical Center, Geert Grooteplein-Zuid 26-28, 6525 GA Nijmegen, The Netherlands; b Department of Biochemistry, Netherlands Cancer Institute, Plesmanlaan 121, 1066 CX Amsterdam, The Netherlands

**Keywords:** peptide conformation, *cis* peptide bond, structure validation, structure correction

## Abstract

A coordinate-based method is presented to detect peptide bonds that need correction either by a peptide-plane flip or by a *trans*–*cis* inversion of the peptide bond. When applied to the whole Protein Data Bank, the method predicts 4617 *trans*–*cis* flips and many thousands of hitherto unknown peptide-plane flips. A few examples are highlighted for which a correction of the peptide-plane geometry leads to a correction of the understanding of the structure–function relation. All data, including 1088 manually validated cases, are freely available and the method is available from a web server, a web-service interface and through *WHAT_CHECK*.

## Abstract 2

A method is presented to detect peptide bonds that need either a *trans*–*cis* flip or a peptide-plane flip.

## 1. Introduction   

Peptide bonds connect adjacent amino acids in proteins. The partial double-bond character of the peptide bond restricts its torsion. The dihedral angle ω (C^α^_*i*−1_—C_*i*−1_—N_*i*_—C^α^_*i*_) typically has values around 180° (*trans*) or 0° (*cis*), although exceptions are possible (Berkholz *et al.*, 2012 ▸). The C^α^_*i*–1_—C^α^_*i*_ distance is around 3.81 Å in the *trans* conformation and around 2.94 Å in the *cis* conformation. The *trans* conformation is energetically preferred over the *cis* conformation owing to unfavourable nonbonded interactions between the two C^α^—H^α^ moieties flanking the peptide bond (Zimmerman & Scheraga, 1976 ▸; Stewart *et al.*, 1990 ▸ and references therein; Jabs *et al.*, 1999 ▸ and references therein). The *cis* imide bond *X*—Pro is more frequently observed than the *cis* amide bond *X*—Xnp (where Xnp is any residue except Pro) because the C^α^_*i*−1_ and O_*i*−1_ atoms have similar third neighbours in either *X*—Pro conformation (Ramachandran & Sasisekharan, 1968 ▸).

In the early days of protein crystallography *cis* peptides were almost completely absent in the available protein crystal structures (Ramachandran & Mitra, 1976 ▸). Therefore, characterization of *cis*-peptide geometry in protein crystal structures was very difficult. In the early 1990s the number of structures deposited in the PDB allowed the first studies of the local protein environment of *cis* peptides. Stewart *et al.* (1990 ▸) characterized 17 *cis* *X*—Xnp and 99 *cis* *X*—Pro peptides and MacArthur & Thornton (1991 ▸) analysed 58 *cis* *X*—Pro residues in a set of nonhomologous structures. Many surveys showed that the computationally derived energy differences between the *cis* and *trans* isomers are only partially reflected in their frequency of occurrence in the PDB. Jabs and coworkers reviewed the arguments for the rarer than expected occurrence of *cis* peptide bonds in protein structures (Jabs *et al.*, 1999 ▸). These arguments were mainly related to protein energetics and to protein function, as several studies had observed *cis* peptides at functionally important locations such as active sites or protein binding interfaces (Stoddard & Pietrokovski, 1998 ▸ and references therein; Weiss *et al.*, 1998 ▸ and references therein). Several studies showed a correlation between the resolution of the crystal structure and the number of *cis* peptides (Stewart *et al.*, 1990 ▸; Weiss *et al.*, 1998 ▸; Pal & Chakrabarti, 1999 ▸). Several authors, including Jabs and coworkers, have noted that the underrepresentation of *cis* peptides is partly the result of the *a priori* assumption often made upon determining X-ray crystal structures that all peptides have a *trans* conformation (Huber & Steigemann, 1974 ▸; Stewart *et al.*, 1990 ▸; Weiss *et al.*, 1998 ▸; Jabs *et al.*, 1999 ▸). In the 1970s it had already been noted that reinterpretation of electron-density maps might reveal many previously unnoticed *cis* peptides in protein structures (Huber & Steigemann, 1974 ▸; Ramachandran & Mitra, 1976 ▸).

**Figure 1:**
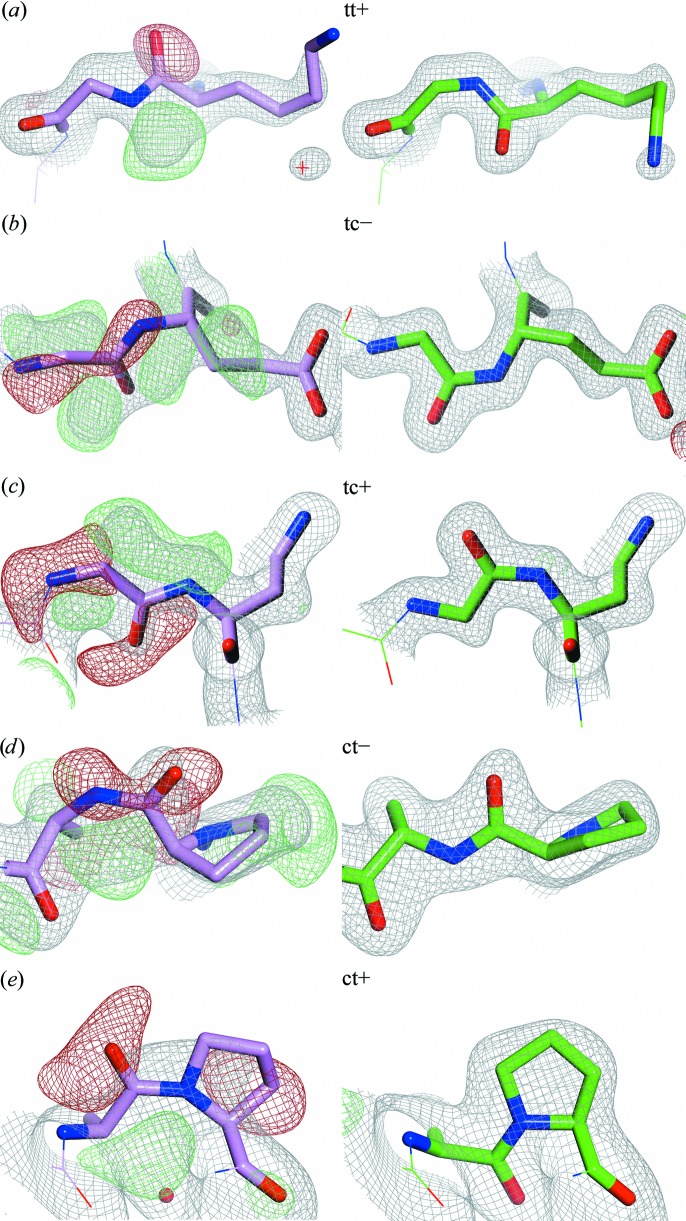
Representative peptide flips. The left figures show the peptide conformation found in the PDB (pink structures) and electron-density maps obtained from the Uppsala Electron Density Server (Kleywegt *et al.*, 2004 ▸). The figures on the right show the conformation and electron density of the corresponding re-refined structure models (green) from the PDB_REDO databank (Joosten *et al.*, 2011 ▸). The flip type is indicated (tt− and cc− are not flips and thus are not illustrated). A cc+ example could not be found in the PDB. (*a*) Gly90, chain *A*, PDB entry 3hr7 (Cheng *et al.*, 2012 ▸), 1.8 Å resolution. (*b*) Glu175, chain *C*, PDB entry 3k2g, 1.8 Å resolution. (*c*) Gln541, chain *A*, PDB entry 1w0o (Moustafa *et al.*, 2004 ▸), 1.9 Å resolution. (*d*) Ala82, chain *A*, PDB entry 1v6i (Kundhavai Natchiar *et al.*, 2004 ▸), 2.15 Å resolution. (*e*) Pro55, chain *B*, PDB entry 1j1j (Sugiura *et al.*, 2004 ▸), 2.2 Å resolution. The *PDB_REDO* program *pepflip* (Joosten *et al.*, 2011 ▸) performs tt+ peptide-plane flips, which are a combination of both a C=O flip and an N—H flip. Re-refinement alone can lead to N—H flips (tc− and ct− flips) when the signal in the X-ray data is strong enough. Therefore, the net result of a tt+ flip and subsequent refinement may become a tc+ flip. Consequently, *PDB_REDO* is able to correct many (but certainly not all) peptides in need of a *trans*–*cis* flip. The 2*mF_o_* − *DF*_c_ (grey mesh) and *mF_o_* − *DF*_c_ maps (+, green mesh; −, red mesh) have been contoured at 1.5σ and ±3σ, respectively, and have been rendered with a grid size of 0.2 Å for visualization purposes. The figures were prepared with *CCP*4*mg* (McNicholas *et al.*, 2011 ▸).

An incorrectly assigned peptide conformation can be in need of any of a number of possible corrections. The first and most common is a rotation of the entire peptide plane by 180°, referred to as an amide flip (McCammon *et al.*, 1977 ▸), peptide-plane flip (Hayward, 2001 ▸) or peptide flip (Joosten *et al.*, 2011 ▸). In the following these peptide-plane flips are called tt+ [Fig. 1 ▸; the first two symbols are either c for *cis* or t for *trans*, reflecting the situation before and after the flip, respectively; the third symbol is a plus (+) sign if the conformational change includes a flip of the backbone carbonyl C=O, otherwise it is a minus (−) sign]. The other two corrections are *trans*↔*cis* flips that can either constitute a flip of the backbone C=O (tc+ or ct+) or a flip of the backbone amide N—H (tc− or ct−). A correct peptide is either tt− or cc−. Every type of flip was observed in crystal structures deposited in the Protein Data Bank (PDB; Berman *et al.*, 2007 ▸) except cc+ (*cis*-peptide flips that include a flip of the C=O). Fig. 1 ▸ shows representative examples for the five possible codes that include a flip and that have actually been observed in the PDB. More examples can be found on the associated website (http://swift.cmbi.ru.nl/gv/flips/).

Frömmel & Preissner (1990 ▸) were the first to predict *cis* *X*—Pro peptides based on just the amino-acid sequence. Later, the prediction algorithms were expanded to take into account *X*—Xnp peptides (Pahlke *et al.*, 2005 ▸; Exarchos *et al.*, 2009 ▸). Machine-learning approaches have been applied that included multiple sequence alignments, predicted secondary structure and predicted solvent accessibility (Wang *et al.*, 2004 ▸; Song *et al.*, 2006 ▸; Exarchos *et al.*, 2009 ▸). These sequence-based algorithms correctly predict the conformation of about three quarters of the tested peptides. A detailed comparison of these algorithms is impossible since the only software still available today is that of Song *et al.* (2006 ▸). Sequence-based algorithms have probably become obsolete because much higher prediction accuracy is required in the everyday practice of crystallographers.

Jabs and coworkers were the first to discuss geometric aspects that could be used to detect incorrectly modelled *X*—Xnp tc− flips (Jabs *et al.*, 1999 ▸). Their algorithm was the first coordinate-based flip-prediction method that took into account the locally distorted geometrical environment of a misassigned peptide bond. Initially, the algorithm was based on just four *cis* peptides in coagulation factor XIII (Jabs *et al.*, 1999 ▸). This method was implemented in *WHAT IF* but suffered from many false positives, *i.e.* incorrectly predicted *trans*-to-*cis* flips. Weiss and Hilgenfeld (WH) later refined their algorithm using a set of 17 incorrectly assigned *trans* peptides (Weiss & Hilgenfeld, 1999 ▸). This method was implemented for comparison purposes, but it was found to give many false-negative predictions, *i.e.* it overlooked necessary tc− flips (see Table 5). The WH method was designed for *X*—Xnp peptides only. A structure-based algorithm predicting *trans*-to-*cis* flips in *X*—Pro peptides does not yet exist. *Cis*-to-*trans* flips are much rarer than *trans*-to-*cis* flips, mostly as a consequence of the *a priori* assumption that peptides are in the *trans* isomer. *Cis*-to-*trans* flips have not been predicted by any algorithm known to date.

With the introduction of *PDB_REDO* (Joosten & Vriend, 2007 ▸; Joosten *et al.*, 2009 ▸), it became possible to reinterpret experimental X-ray data in an automated way, and when Joosten and coworkers developed a method called *pepflip*, peptide-plane tt+ flips could be detected and corrected based on the fit to the local electron density (Joosten *et al.*, 2011 ▸). It should be noted, however, that *pepflip* does not perform *trans*↔*cis* flips, and occasionally performs a tt+ flip when actually a *trans*–*cis* flip is needed.

All crystallographic PDB structures were compared with their PDB_REDO counterparts and many examples were observed of the different flip types in Fig. 1 ▸. With the large set of peptide flips in hand, we asked whether these flips could be used to obtain a large training set for a Random Forest (RF; Breiman, 2001 ▸) machine-learning approach for the structure-based prediction of peptide-plane inversions. The method predicts 70 461 peptide-plane flips and 4617 *trans*–*cis* flips in the PDB.

## 2. Methods   

### 2.1. Data selection   

Pairs of X-ray structures were obtained from the PDB and PDB_REDO releases of 20 October 2014 and were used only if they met the selection criteria listed in Table 1 ▸.

From these PDB files, stretches of four residues were selected if they met the selection criteria listed in Table 2 ▸.

The tetrapeptides in the data set were divided into *X*-*X*-Pro-*X*, *X*-*X*-Gly-*X* and *X*-*X*-Xnpg-*X* (where Xnpg is any residue except for Pro or Gly), which, for brevity, are called *X*-Pro, *X*-Gly and *X*-Xnpg, respectively.

**Figure 2:**
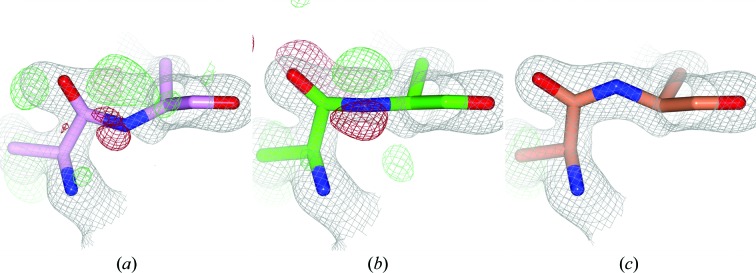
Stepwise improvements from *trans* to *cis*. (*a*) The peptide bond between Arg71 and Phe72 (ω = 132°) in PDB entry 2bmd (Huber & Scheidig, 2005 ▸) needs a tc− flip. (*b*) The peptide has been flipped only ‘halfway’ in PDB_REDO (ω = 81°). (*c*) The fully flipped and refined *cis* peptide (ω = 9°). The side chains have been omitted beyond C^β^ for clarity. Maps are as in Fig. 1 ▸.

A large number of tetrapeptides were manually validated. These tetrapeptides represented both correct and incorrect conformations. 173 peptides in 81 PDB_REDO files were rebuilt and re-refined because visual inspection suggested that the PDB_REDO conformation was not plausible (see, for example, Figs. 2 and 4). This resulted in a validation data set consisting of 1088 tetrapeptides (see Table 3 ▸) in 438 PDB structures, 192 of which contained at least one genuine flip. Many more peptides were inspected, but were not included because the quality of the electron density was not good enough. The validation data were gathered over the course of this study. Many tetrapeptides were expected to be difficult for an automated method to predict correctly. These difficult cases were deliberately added to the validation set and included not only incorrect tetrapeptides but also correct ones. About 400 cases were included that had been incorrectly classified by earlier versions of the classification algorithm developed here or by the first Jabs, Weiss and Hilgenfeld algorithm.

A special menu was added to *WHAT IF* (Vriend, 1990 ▸) that compares PDB entries with their PDB_REDO mates. Options in this menu allow the detection of many differences in coordinates, angles, torsion angles, *B* factors *etc.* between PDB and PDB_REDO pairs. The *WHAT IF* procedure that compares the peptide conformations in the ∼71 000 PDB–PDB_REDO pairs of protein structures and that assigns the flip types was based on three variables describing the difference between the central peptide planes in the corresponding tetrapeptides: the (C=O, C=O) angle, the (N—H, N—H) angle and the ω torsion-angle difference. The training examples were taken from structures solved at 3.5 Å resolution or better, except for the examples for peptide-plane flips because validation of flip assignment and prediction was found to be more accurate when only structures solved at 2.2 Å resolution or better were included. In total, at least one clearly flipped peptide was observed in 16 688 PDB_REDO entries.

Structures were rebuilt manually with *Coot* (Emsley *et al.*, 2010 ▸) and re-refined with *REFMAC* (Murshudov *et al.*, 2011 ▸). The refinement strategy and parameters were obtained from the *PDB_REDO* protocol (Joosten *et al.*, 2012 ▸). The *CCP*4 (Winn *et al.*, 2011 ▸) program *EDSTATS* (Tickle, 2012 ▸) was used to calculate real-space correlation coefficients.

### 2.2. Prediction   

For each tetrapeptide a large number of features was calculated using *WHAT IF*, including C^α^—C^α^ distances, C^β^—C^β^ distances, O—O distances, backbone torsion angles, backbone bond lengths, backbone bond angles up to C^β^ atoms, chiral volumes, C—O—C—O angles, the O_*i*−1_ bump score and the carbonyl alignment with an α-helix nearby in the sequence, three-state secondary structure as derived from *DSSP* (Kabsch & Sander, 1983 ▸; Touw *et al.*, 2015 ▸) and *B* factors from BDB entries (Touw & Vriend, 2014 ▸), which consistently have full isotropic *B* factors, unlike PDB entries that can have residual *B* factors from TLS (Schomaker & Trueblood, 1968 ▸) refinement. The WH method was implemented in *WHAT IF* as described by Weiss & Hilgenfeld (1999 ▸). The WH ‘penalty-function score’ (*D*_tot_) is also one of its features. Random Forest (Breiman, 2001 ▸) classifiers were constructed using the *R* (R Core Team, 2015 ▸) package *randomForest* (Liaw & Wiener, 2002 ▸) and tuned using repeated fivefold cross-validation. The classifier objects were automatically converted into Fortran code for inclusion in *WHAT IF* and *WHAT_CHECK* (Hooft *et al.*, 1996 ▸).

## 3. Results   

### 3.1. Peptide-plane inversion examples   

Thousands of peptide-plane inversions were observed by comparing PDB structures with their PDB_REDO counterparts (Table 3 ▸).

Visual inspection of many peptide-plane inversions indicated that about 90% of the flips introduced by *PDB_REDO* are correct. Sometimes the *PDB_REDO* peptide conformation is suboptimal. In some of the tc− cases, for example, the ω angle can end up at around 90° in the *PDB_REDO* output model (Fig. 2 ▸). These conformations, which are essentially halfway between the wrong and the right conformation, are the result of *trans*-peptide restraints outweighing the crystallographic data during refinement. The problems can be resolved by additional refinement with *cis*-peptide restraints.

### 3.2. Prediction of peptide flips   

The studies by Weiss & Hilgenfeld (1999 ▸), and our own visual inspection of hundreds of peptide planes that needed a flip to better agree with the X-ray data, revealed that a number of geometric variables tend to deviate from their common values when a peptide plane has been built in the wrong conformation. For instance, the angle C^α^_*i*−1_—C_*i*−1_—N_*i*_ tends to be smaller than normal for *X*-Xnpg tc− peptides, and the *B* factor of the O atom in the plane tends to be high if the peptide plane needs a tt+ flip. Therefore, all features were collected that could possibly characterize the local distortion of an incorrectly modelled peptide plane. These features were not limited to geometric variables and *B* factors, but also included secondary structure and a description of the environment of the O atom in the peptide plane. Other variables such as the hydrogen-bonding status and rotamericity of the side chains can be added, but they are computationally intensive and, for reasons that we do not yet fully understand, do not influence the prediction accuracy of the method very much. A comprehensive list of variables is available on the project’s website. For each flip type a classifier was trained to determine variable combinations that can separate peptides in need of a flip from correct peptides. The flip-type specific classifiers were combined into one classifier per residue class (*X*-Xnpg, *X*-Pro and *X*-Gly). All classifiers were validated using an independent test set. Table 3 ▸ shows the number of peptides in the training test sets. Classifiers for *cis*-to-*trans* flips and other small categories were not constructed because classifiers fitted to too few training examples will not be generally applicable. The full details of the design, implementation and use of the classifiers for the four situations for which adequate data was available and the manual decision tree for *X*-Pro tc− are given on the project’s website.

Table 4 ▸ lists the results for the four residue and flip-type specific RF classifiers. The combined classifiers predict *X*-Xnpg flip types (tt−, tt+, tc−, tc+) with an accuracy of 93%. This includes all 12 tc+ cases that were found in the PDB and that were not in the training set. The accuracy is 95% without these *X*-Xnpg tc+ cases. *X*-Pro tt−, tt+, tc− and tc+ flips in the test set can be classified with an overall accuracy of 93%.

#### 3.2.1. *X*-Xnpg tc−   

Table 5 ▸ shows the prediction outcome in terms of true positives (TP), true negatives (TN), false positives (FP) and false negatives (FN) for the *X*-Xnpg tc− and tt− test peptides. The confusion tables for the results obtained with the WH method are also shown. Weiss & Hilgenfeld (1999 ▸) mentioned that their cutoff could not be validated, as the experimental data for most of the structures in their data set were not available. The optimal cutoff for the WH *D*_tot_ score could be determined using the 539 cases that were manually validated using electron density. With the new threshold both the TP and FN rates improved more than 75% compared with the original WH threshold (Table 5 ▸). The RF-based method further increased this performance by decreasing the FP rate at the cost of a small increase in the FN rate. Note that a low FP rate is more important for protein structure-validation purposes than for prediction-assisted rebuilding and re-refinement.

The variables that were most important for separating *X*-Xnpg tc− from tt− were also used in the WH algorithm: ϕ_i_, the backbone angles O_*i*−1_—C_*i*−1_—N_*i*_, C^α^_*i*−1_—C_*i*−1_—N_*i*−1_, C^α^_*i*−1_—C_*i*−1_—O_*i*−1_ and C_*i*−1_—N_*i*_—C^α^_*i*_, and the C^α^_*i*−1_—C^α^_*i*_ distance. In addition, the C^α^_*i*_—C_*i*_—N_*i*_ angle, the C^α^_*i*_ chiral volume and the C^β^_*i*−1_—C^β^_*i*_ distance were found to be important for the RF method. Other WH bond lengths and angles were found to be less important. The full list of variables and their importance can be found on the associated website. In general, and not unexpectedly, the variables extracted from the inner two residues in tetrapeptides contributed most to the prediction accuracy of all flip types.

Application of the *X*-Xnpg tc− method to *X*-Gly cases did not reveal any new *X*-Gly tc− flips. This result suggests that the method might not be able to detect *X*-Gly tc− flips; after all, it was trained only on *X*-Xnpg tetrapeptides. Another explanation is that *X*-Gly tc− flips are simply very rare. This explanation is supported by the observation that a *cis* peptide modelled in the *trans* conformation is more easily corrected to the *cis* conformation automatically during refinement when the residue type is Gly rather than any other type.

#### 3.2.2. *X*-Pro tc+   

**Figure 3:**
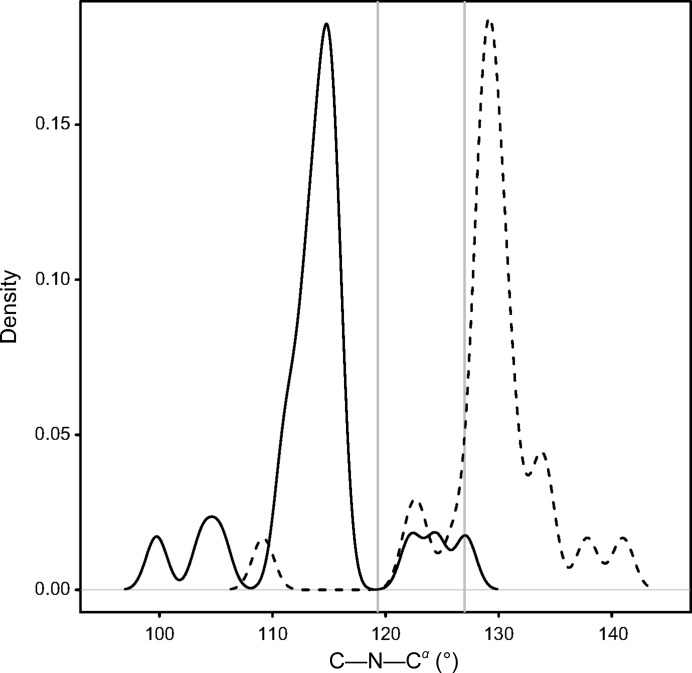
The C_*i*−1_—N_*i*_—C^α^_*i*_ angle before and after correction of *X*-Pro tc+ cases. The curved lines show Gaussian kernel density estimates for the C_*i*−1_—N_*i*_—C^α^_*i*_ backbone angle for 25 *X*-Pro tc+ cases in the test set before (solid line) and after (dashed line) correction and re-refinement. The vertical lines show the values for *trans*-Pro (119.3 ± 1.5°) and *cis*-Pro (127.0 ± 2.4°) reported by Engh & Huber (2001 ▸).

*X*-Pro tc+ cases in the test set could be classified without any FP. Important variables are the angle between the carbonyl of the central peptide bond and the carbonyl before that, the C_*i*−1_—N_*i*_—C^α^_*i*_ and N_*i*−1_—C^α^_*i*−1_—C_*i*−1_ angles, ψ_*i*−1_, the C^α^_*i*−1_—C^α^_*i*_ distance and the O_*i*−1_ bump score. Engh & Huber (2001 ▸) observed that the bimodal distribution of C_*i*−1_—N_*i*_—C^α^_*i*_ in high-resolution peptide fragments was caused by differences between the *cis* and *trans* forms. The median C_*i*−1_—N_*i*_—C^α^_*i*_ angle for tc+ cases in the test set (116.0°) was smaller than the median for tt− cases in the PDB (121.0°) and the value Engh & Huber (2001 ▸) reported for *trans* proline (119.3 ± 1.5°), but after re-refinement the median (129°) was just above the value that Engh and Huber reported for *cis* proline (127.0 ± 2.4°). This is illustrated in Fig. 3 ▸. The figures on the website show the change in all variables before and after correction and re-refinement of the tetrapeptides in the test set.

#### 3.2.3. *X*-Xnpg tt+   

The 480 *X*-Xnpg tt+ cases in the test set could be classified with seven FP and three FN. One FN and one FP are next to a *trans*–*cis* flip. The eight *X*-Xnpg cases in the test set that *PDB_REDO* failed to flip were correctly predicted by the RF method. The most important variables for predicting *X*-Xnpg tt+ cases are the* B* factors of the O and C atoms in the central peptide plane, ϕ, ψ, the secondary structure and the C^β^_*i*−1_—C^β^_*i*_ distance.

#### 3.2.4. *X*-Gly tt+   

The *B* factor of the central O atom is also very important for *X*-Gly classification, as are the C_*i*−1_ *B* factor, the C_*i*−1_—N_*i*_—C^α^_*i*_ and N_*i*−1_—C^α^_*i*−1_—C_*i*−1_ angles and the secondary structure of residue *i* − 1. Gunasekaran *et al.* (1998 ▸) studied *in vivo* conversion between type I and type II β-turns and between type I′ and type II′ β-turns. They reported the importance of the *B*-factor distribution of the central O atom in flippable β-turns (Gunasekaran *et al.*, 1998 ▸). The best classification results were obtained for *X*-Gly (two FP and one FN) when the tt+ classifier was trained with data from structures solved at a resolution better than 2 Å, probably because the backbone is generally less well defined in low-resolution structures, resulting in higher *B* factors caused by the low resolution rather than by a peptide in need of a flip. The inherent mobility of Gly may also explain the fact that many surface-located *X*-Gly were found that could not be interpreted well because of electron density that was too poor.

#### 3.2.5. *X*-Pro tc−   

**Figure 4:**
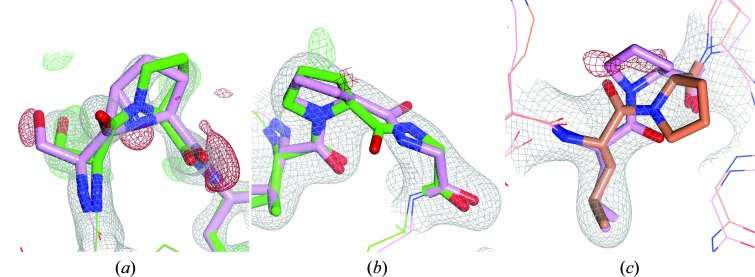
*X*-Pro problems characterized by positive ϕ_*i*_ angles. (*a*) *X*-Pro tc− flip; Ser339-Pro340, chain *A*, PDB entry 1se6 (Zhao *et al.*, 2005 ▸), 1.75 Å resolution. (*b*) Pro-Gly tt+ flip; Val53-Pro54-Gly55, chain *A*, PDB entry 1hxd (Weaver *et al.*, 2001 ▸), 2.40 Å resolution. (*c*) *X*-Pro tt+ flip; Leu203-Pro204, chain *B*, PDB entry 1cdd (Almassy *et al.*, 1992 ▸), 2.80 Å resolution. Electron-density maps were calculated using the PDB structure and are rendered as in Fig. 1 ▸. The PDB structures are shown in pink and the PDB_REDO structures in green; the manually corrected and re-refined structure is shown in orange.

All 40 *X*-Pro tc− in the test set derived from the PDB–PDB_REDO comparison had a positive ϕ_*i*_, while the ϕ_*i*_ for tt− and tc+ cases is always around −60°. Remarkably, in the entire set of crystal structures with deposited structure factors 904 *X*-Pro cases were found with a positive ϕ_*i*_, 86 of which were in structures solved at a resolution of between 1.2 and 2.0 Å. These 904 cases all were either *X*-Pro tc− flips (Fig. 4 ▸ *a*) or *trans* *X*-Pro with an otherwise incorrect nitrogen chirality (‘NCh’).

The ‘NCh’ class includes tetrapeptides where residue *i* + 1 needs a tc+ flip (*e.g.* His173-Leu174-Pro175-Pro176 in PDB entries 1bug and 1bt2; Klabunde *et al.*, 1998 ▸) or a tt+ flip [*e.g.* Thr52-Val53-Pro54-Gly55 (see Fig. 4 ▸ *b*) in chain *A* of PDB entry 1hxd (Weaver *et al.*, 2001 ▸) and Gly152-Ala153-Pro154-Gly155 in chain *B* of PDB entry 4le4 (T. Jiang, H.-C. Chan, C.-H. Huang, T.-P. Ko, T.-Y. Huang, J.-R. Liu & R.-T. Guo, unpublished work)]. Surprisingly, one of the examples was even an *X*-Pro tt+ flip (Pro204 in chain *B* of PDB entry 1cdd; Almassy *et al.*, 1992 ▸; Fig. 4 ▸ *c*). The ‘wrinkled’ tc− prolines with positive ϕ_*i*_ often have an almost straight C_*i*−1_—N_*i*_—C^α^_*i*_ angle (Fig. 4 ▸ *a*), which is probably the result of very tight ω restraints, and are reminiscent of the intermediate structure of the *trans*-to-*cis* transition of Gly78-Ile79 observed during the refinement of rubrerythrin (Stenkamp, 2005 ▸). From the 904 cases, 59 tc− flips and 22 ‘NCh’ *X*-Pro cases were visually inspected. If the angle τ (N_*i*_—C^α^_*i*_—C_*i*_) is larger than 112.5° and the bump score of the O atom in the peptide plane is larger than 0.26 *WHAT IF* bump score units, then the *X*-Pro with a positive ϕ_*i*_ is not a tc− peptide but an ‘NCh’ *X*-Pro. This rule predicts 404 *X*-Pro tc− flips and 500 ‘NCh’ *X*-Pro.

### 3.3. *Cis*→*trans* flips   

44 clear *cis*-to-*trans* flips have been found in this study. For *trans* *X*-Xnpg tetrapeptides modelled as *cis* tetrapeptides the median C^α^_*i*−1_—C^α^_*i*_ distance (3.34 Å) tends to be larger than the median C^α^_*i*−1_—C^α^_*i*_ distance for correct *cis* *X*-Xnpg tetra­peptides (2.95 Å). Similarly, the median C_*i*−1_—N_*i*_—C^α^_*i*_ angle (131°) tends to be larger than normal (125°). The median C^α^_*i*−1_—C^α^_*i*_ distance (3.55 Å) and C_*i*−1_—N_*i*_—C^α^_*i*_ angle (159°) for ct− and ct+ *X*-Pro tetrapeptides also tend to be larger than normal (2.95 Å and 127°, respectively).

### 3.4. Molecular replacement   

Molecular replacement (MR) using a *trans* peptide is a very common reason for failing to model a *cis* peptide correctly. This section describes a few examples of this problem.

In the *Escherichia coli* family 31 α-glycosidase Yicl, Cys316 and Val477 adopt a *cis* conformation in both the free form (PDB entries 1xsi and 1xsj; Lovering *et al.*, 2005 ▸) and when bound to the sugar adduct eq-5-fluoroxylosyl (PDB entry 1xsk; Lovering *et al.*, 2005 ▸). The authors listed both residues as part of the active site of the α-glycosidase and mentioned that *cis*-Cys316 orients the side chain of Trp315 to direct Cys307 toward the sugar-binding site (Lovering *et al.*, 2005 ▸). Notably, PDB entry 1xsi was the MR search model for PDB entries 1xsj and 1xsk, but in this process the one Cys316 in chain *A* that was correctly in the *cis* conformation became *trans* in the latter two structures. Val477 was in the incorrect *trans* conformation in all six chains related by noncrystallographic symmetry (NCS) in each of the three PDB structures.

The PDB_REDO structure of PDB entry 1uyq (P. Isorna, J. Polaina & J. Sanz-Aparicio, unpublished work) clearly showed that a tc− flip should be performed for Ser399. The same flip was predicted in all β-glucosidase A molecules listed in the PDB file as related structures [PDB entries 1bga and 1bgg (Sanz-Aparicio, Hermoso, Martínez-Ripoll, Lequerica *et al.*, 1998 ▸) and 1tr1 and 1e4i (Sanz-Aparicio, Hermoso, Martínez-Ripoll, González *et al.*, 1998 ▸)]. However, the structure factors are not available for any of these structures. Although there is no paper to support it, it seems very likely that one of the related structures was used as a search model to solve the structure 1uyq and Ser399 should be *cis* in all related structures.

Even though it is more likely that a *cis* peptide will be accidentally refined as a *trans* peptide, *cis*-to-*trans* flips were observed in four different chains of PDB entry 1v6i (Kundhavai Natchiar *et al.*, 2004 ▸) at residues Lys77-Asp78 and one additional ct− flip only in chain *A* at Pro81-Ala82. The structure was solved at 2.15 Å resolution using MR with PDB entry 2pel (Banerjee *et al.*, 1996 ▸) as a starting structure. However, the corresponding residues in PDB entry 2pel all have the correct *trans* conformation. It is therefore unclear to us how the *cis* peptides have been introduced into the 1v6i model.

### 3.5. Crystallographic improvement   

**Figure 5:**
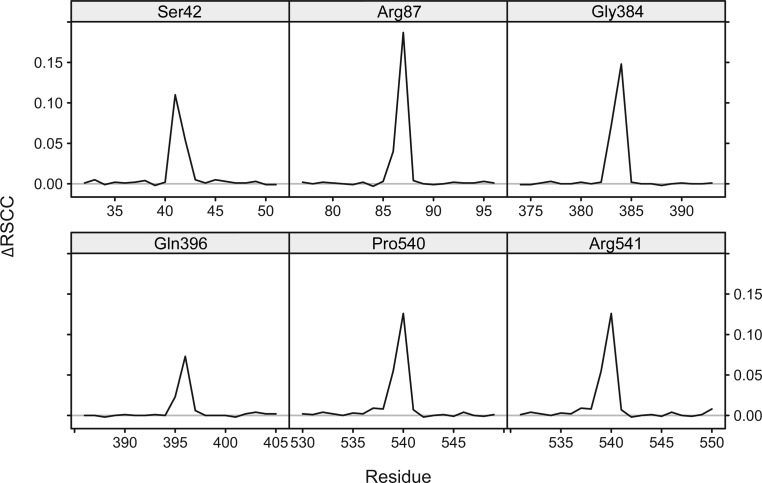
Improvement of the real-space correlation coefficient (RSCC) in PDB entry 2z81 (Jin *et al.*, 2007 ▸; 1.80 Å resolution) after rebuilding and re-refining incorrect peptide bonds. The panels show for six peptide bonds in a region of 20 surrounding residues the increase in average backbone-atom RSCC (including C^β^) when the peptides are corrected and the structure is re-refined, compared with re-refinement of the structure only. The residue after the central peptide bond is indicated in the top bar. Pro540 was corrected by a tc+ flip and all other peptides by tt+ flips. Re-refinement alone already resulted in correction of the conformation of Arg541. Re-refinement details and figures showing the local backbone of the peptide bonds can be found on the associated website.

The real- and reciprocal-space correlation of several representative corrected and re-refined PDB structure models was analysed to investigate the effect on crystallographic quality metrics of flip correction and re-refinement. As an example, the local improvement after a flip in terms of fit to the electron density is shown for PDB entry 2z81 (Jin *et al.*, 2007 ▸) in Fig. 5 ▸. Similar figures for the other re-refinement examples can be found on the website.

The improvement in *R*_work_/*R*_free_ as a result of flipping and re-refining was 0.14/0.41% with respect to re-refining only. The work/free reciprocal-space correlation improvement was 0.08/0.22%. The largest improvement in *R*_work_/*R*_free_ (0.23/0.43%) and work/free reciprocal-space correlation (0.18/0.28%) was found for PDB entry 1hi8 (Butcher *et al.*, 2001 ▸), in which 16 flips were necessary. Although the global refinement metrics improve only marginally, the local metrics show a clear improvement upon flipping and, more importantly, sometimes a flip alters our understanding of the relationship between structure and function of a protein.

### 3.6. Biological implications of newly detected flips   

#### 3.6.1. Rab4a   

**Figure 6:**
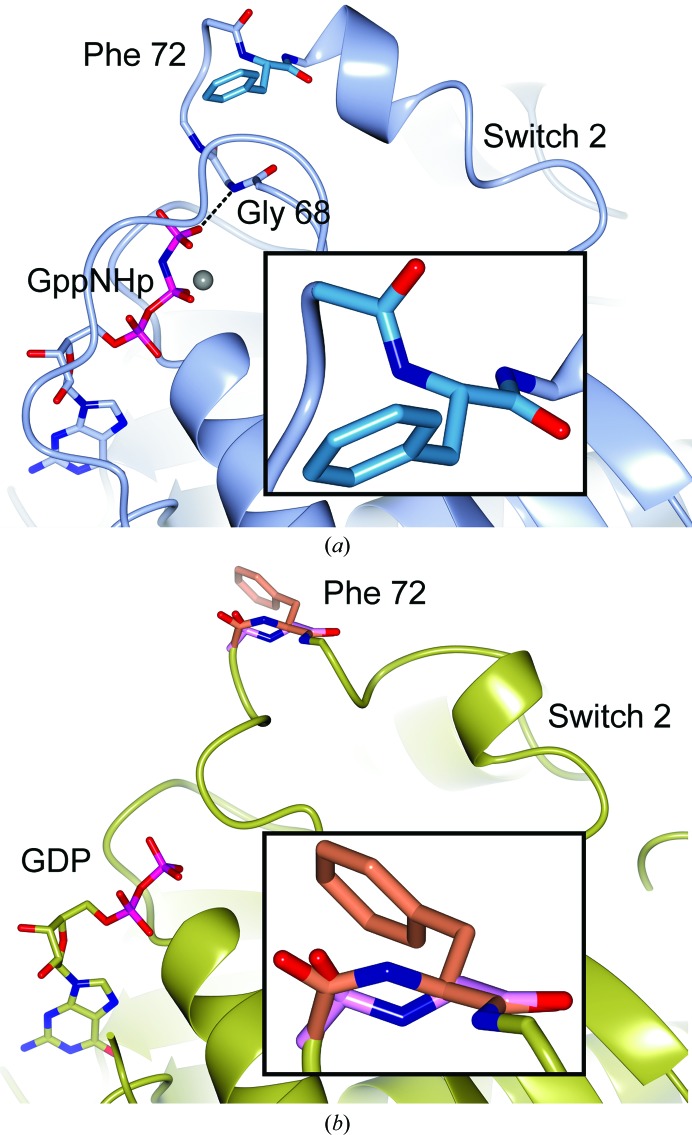
Rab4a. (*a*) The active state (PDB entry 2bme; Huber & Scheidig, 2005 ▸) with *trans*-Phe72 (inset). The grey sphere is magnesium. (*b*) The inactive state (PDB entry 2bmd; Huber & Scheidig, 2005 ▸) with the corrected and re-refined *cis*-Phe72 in orange (inset). The incorrect *trans*-Phe72 backbone is shown in pink.

A tc− flip was predicted for Phe72 in the GDP-bound state of human Rab4a (PDB entry 2bmd). This residue is referred to as Phe70 in the associated paper (Huber & Scheidig, 2005 ▸). Phe72 is located at the start of α-helix H2 in the switch 2 region of the small GTPase (Fig. 6 ▸). Fig. 2 ▸ shows Phe72 in the GDP-bound Rab4a before and after correction and re-refinement.

In the GppNHp-bound Rab4a (PDB entry 2bme; Huber & Scheidig, 2005 ▸) Phe72 has the *trans* conformation (Fig. 6 ▸ *a*). Upon GTP hydrolysis, the hydrogen bond between the γ-phosphate and Gly68 is lost and conformational rearrangements take place in the switch 2 region (Huber & Scheidig, 2005 ▸). We cannot exclude that the *cis*-form was selected during crystallization, but our findings could also suggest that Arg71-Phe72 *trans*–*cis* isomerization might be part of this rearrangement (Figs. 2 ▸ and 6 ▸ *b*), which would indicate that the re­arrangement process might be more complicated than previously thought. Phe72 is 97% conserved in the HSSP alignment (Touw *et al.*, 2015 ▸) and is part of the homologous effector-binding epitope of Rab5a (Huber & Scheidig, 2005 ▸), suggesting a role in discrimination between different effector proteins.

#### 3.6.2. Inosine 5′-monophosphate dehydrogenase   

**Figure 7:**
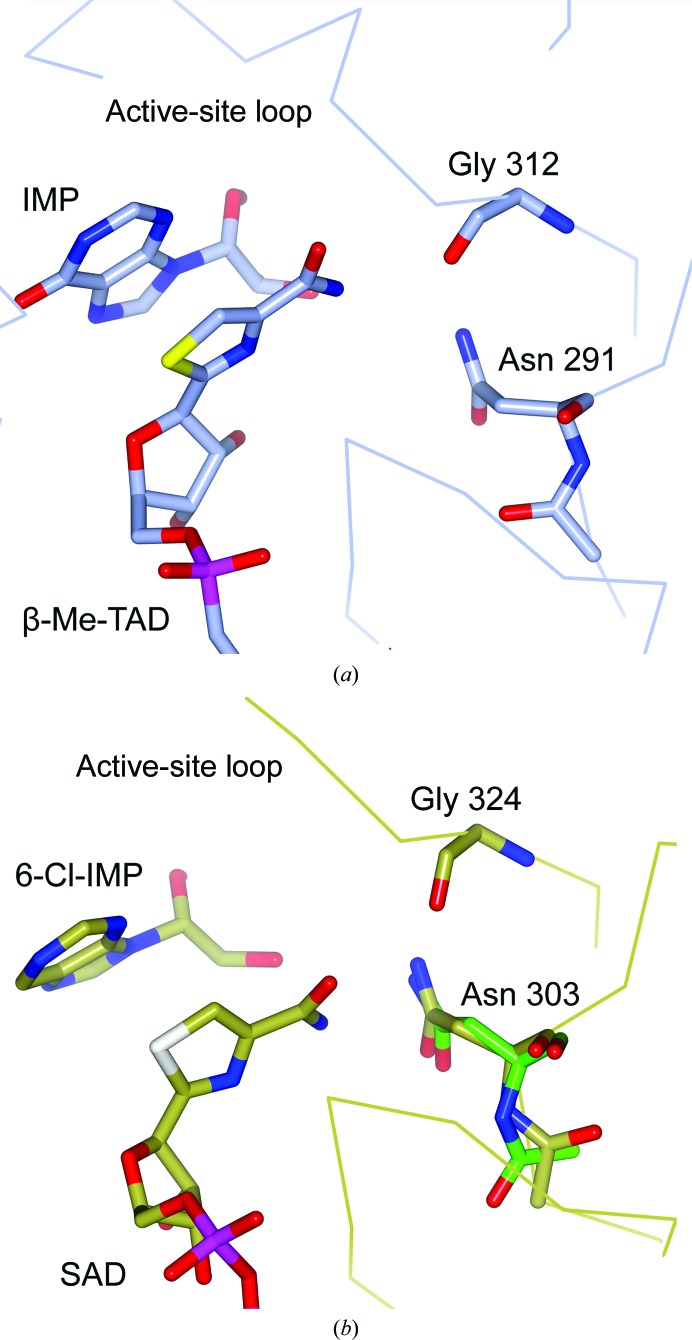
Part of the active site of inosine 5′-monophosphate (IMP) dehydrogenase (IMPDH). (*a*) *T. foetus* IMPDH (PDB entry 1lrt; Gan *et al.*, 2002 ▸). (*b*) Human IMPDH (PDB entry 1b3o; Colby *et al.*, 1999 ▸). The correct *cis*-Asn303 from PDB_REDO is shown in green. The NAD^+^ analogue is β-­methylene thiazole-4-carboxamide adenine dinucleotide (β-Me-TAD) in PDB entry 1lrt and selenazole-4-carboxamide adenine dinucleotide (SAD) in PDB entry 1b3o. The IMP analogue 6-chloropurine riboside 5′-­monophosphate (6-Cl-IMP) is covalently bound to Cys331 in PDB entry 1b3o. The wires trace the C^α^ atoms. Water molecules have been omitted for clarity.

A tc− flip is predicted for Asn291 (Fig. 7 ▸ *a*) in all four copies of *Tritrichomonas foetus* inosine 5′-monophosphate dehydro­genase (IMPDH; PDB entry 1lrt; Gan *et al.*, 2002 ▸). Structure factors were not deposited for PDB entry 1lrt. We nevertheless believe that Asn291 adopts the *cis* conformation because the corresponding peptide plane in the MR search model (PDB entry 1ak5; Whitby *et al.*, 1997 ▸) should also be *cis*. Further, the homologous Asn in human type II IMPDH (PDB entry 1b3o; Colby *et al.*, 1999 ▸) should be *cis* as well (Fig. 7 ▸ *b*).

Asn91 is part of the β-Me-TAD binding site. The Asn291 side chain hydrogen-bonds to the conserved Gly312 carbonyl in the so-called active-site loop and is located less than 4 Å away from the carboxamide of β-Me-TAD (Fig. 7 ▸ *a*). The authors write that the interactions between the active-site loop and this carboxamide are the ‘most striking feature of the ternary complex’ (Gan *et al.*, 2002 ▸). They also write that the homologous Asn in PDB entry 1b3o directly hydrogen-bonds to the carboxamide (Fig. 7 ▸ *b*). The authors extensively allude to the importance of Asn291 in the binding differences of the two ligands. They however fail to notice the peptide-plane flip between PDB entries 1b3o and 1lrt and that in both structures Asn291 is most likely to be a *cis* peptide, a biological feature that in an active site surely is of importance.

## 4. Discussion   

**Figure 8:**
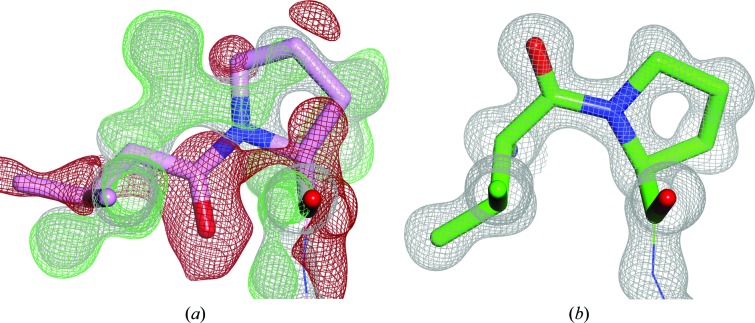
*X*-Pro C=O flip at atomic resolution. The central peptide Val129-Pro130 is shown in (*a*) the 1.2 Å resolution PDB entry 4gqr (Williams *et al.*, 2012 ▸) and (*b*) the corresponding PDB_REDO structure. Note that in the PDB_REDO conformation not only the local backbone, but also the Val (C^γ1^ pointing towards the reader) and Pro side chains fit the density much better. Colours and maps are as in Fig. 1 ▸.

The present study shows that there is a great need for algorithms that can point out peptide bonds that might need flipping and require a crystallographer’s attention. The usefulness of such algorithms is not limited to lower resolution, as flips are sometimes needed at atomic resolution as well (Fig. 8 ▸).

The validation set is not free of selection bias. The residue composition in the validation set has not been matched to the PDB-wide average nor to the training-set average. As mentioned before, the validation set contains relatively difficult cases. Therefore, the ‘true’ performance of the method is presumably even better than the performance reported in Table 4 ▸.

The *PDB_REDO* rebuilding stage explicitly checks whether the real-space correlation of a peptide plane is better before or after a peptide-plane flip (Joosten *et al.*, 2011 ▸). In cases where a tc+ flip is actually needed, a tt+ flip often still fits the density better than no flip at all (see Fig. 1 ▸). Further refinement can often lead to the additionally required N—H flip. This explains why *PDB_REDO* solves many *trans*–*cis* flip problems using only a peptide-plane flip search algorithm. The RF method was trained using peptides that were flipped by *PDB_REDO* and therefore had never seen any cases that *PDB_REDO* failed to correct. One might therefore expect that the classifier might have been biased towards the training set and might have learned to only recognize incorrect peptide conformations that are correctable by *PDB_REDO*. The independent test set contained 69 manually validated *X*-Xnpg cases that needed a tc− flip in both the PDB structure and the PDB_REDO structure. 63 of these cases were classified correctly, which suggests that the method generalizes sufficiently to augment the *PDB_REDO* process. As new crystal structures are continuously being solved and re-refined iteratively by *PDB_REDO*, the method can easily be iteratively improved as well.

12 of the 14 *X*-Xnpg tc− FN corresponded to cases for which a flip was observed or predicted in an NCS-related chain or in the MR search model [Asn267 in chain *A* and Glu435 in chain *D* of PDB entry 1fwx (Brown *et al.*, 2000 ▸), Ser412 in PDB entries 1q7z and 1q85 (Evans *et al.*, 2004 ▸), Ala458 in PDB entries 1w9b and 1w9d (Bourderioux *et al.*, 2005 ▸) and Asp273 in PDB entries 3fx6 (Wang *et al.*, 2009 ▸) and 3fvl (Wang *et al.*, 2010 ▸)]. These FN will therefore in practice not be a large problem. For example, if *WHAT_CHECK* suggests a flip for the same NCS-related residue in chains *B*, *C* and *D*, the crystallographer will of course also check the residue in chain *A* (*e.g.* Asn267 in PDB entry 1fwx). Conversely, a predicted flip might turn out to be an FP when homologous chains are inspected. The FP Glu277 in arginase 1 (PDB entry 3lp4; Di Costanzo *et al.*, 2010 ▸), for example, is predicted to be tc− in chain *A* but not in chain *B*. The FP His188 in the Y364F mutant of 12-oxophytodienoate reductase 3 (PDB entry 2hs8; Breithaupt *et al.*, 2006 ▸) is not predicted to be a tc− flip in another mutant, the wild type or the MR search model. Similarly, Asn137 in the light chain of the antibody structure of PDB entry 2fbj is tt− in other immunoglobulin light chains and Asp383 in the caspase 8 chain of PDB entry 3h11 (Yu *et al.*, 2009 ▸) is tt− in the MR search model (PDB entry 1i4e; Xu *et al.*, 2001 ▸). *WHAT_CHECK* additionally shows plots in which the backbone torsion angles are compared between NCS-related chains. A peptide flip in just one of the two chains leads to a massive peak in this plot.

As mentioned before, *trans* peptides are energetically favoured over *cis* peptides and model-building software by default attempts to build *trans* peptides first. One could also argue that experimentalists pay more attention to *cis* peptides, if these are recognized as such, than to *trans* peptides. The combination of these arguments results in a very small chance of observing incorrectly modelled *cis* peptides. Indeed, only very few clear *cis*-to-*trans* flips were observed in the present study. The fact that automated re-refinement without rebuilding results in few *cis*-to-*trans* corrections suggests that the crystallographic data seldom indicate a strong preference for the *trans* conformation in these cases. Visual inspection also seems to suggest that true *cis* peptides typically occur in well resolved locations. Croll (2015 ▸) recently reported the high rate of *X*-Xnpg *cis* peptides in the PDB. His study suggests that an exhaustive manual search is likely to identify more *cis*-to-*trans* flips. For example, some structure models in the PDB have an unexpected large number of *cis* peptides in regions with poor density. These are likely to be incorrect, but could not be used to train or validate the RF classifiers. An exhaustive manual search for additional *cis*-to-*trans* flips is beyond the scope of this project, but simply rebuilding all *cis­* peptides in the *PDB_REDO* pipeline is computationally feasible. Many *cis–trans* flips will be available for training RF classifiers when all PDB structures have been re-refined using the new *PDB_REDO* pipeline.

The number of observed flips was very large in some classes, while some flip classes were almost completely absent. To increase the number of observations in the smaller classes, observations were fabricated by automatically performing unnecessary flips and subsequent extensive re-refinement. The simulated peptides resulting from this very time-consuming process unfortunately could not be used to successfully predict peptides of the same class observed in the PDB. The small classes will be monitored and in due course the analysis will be repeated when sufficient examples have become available.

The recommendations given recently by Croll (2015 ▸) will help crystallographers to identify spurious *cis* peptides. The method presented here may help to detect *trans* peptides in need of a flip. We believe that these results can help everyday crystallographic practice if they are used. However, the true solution to the problem of incorrect peptide conformations is the training and good supervision of inexperienced crystallo­graphers.

## 5. Availability   

The peptide-validation method has been implemented in *WHAT_CHECK* (http://swift.cmbi.ru.nl/gv/whatcheck/) and is available as a web server (http://swift.cmbi.ru.nl/servers/html/flpchk.html) and as a web service (http://wiws.cmbi.ru.nl/wsdl/). The functionality to perform flips of the tc− and tc+ type will be added to *PDB_REDO*. The *Coot* visualization scripts for PDB_REDO entries (Joosten *et al.*, 2014 ▸) show peptide-plane flips and *trans*↔*cis* flips since *PDB_REDO* version 5.43.

The details of classifier training, the resulting classifiers, all tetrapeptide data used for training and validation, the details and pseudo-code for comparing peptide conformations and all re-refinement example data are available from the associated website at http://swift.cmbi.ru.nl/gv/flips/. *WHAT IF*, including the PDB–PDB_REDO comparison menu and peptide-validation method, is freely available from http://swift.cmbi.ru.nl/gv/facilities/.

## 6. Conclusion   

When applied to the 46 418 233 peptide planes in the PDB, the method predicts 1527 *X*-Xnpg tc− flips, 53 974 *X*-Xnpg tt+ flips, 517 *X*-Pro tc− flips, 2573 *X*-Pro tc+ flips and 16 487 *X*-Gly tt+ flips. *PDB_REDO* has already corrected ∼14% of the peptide-plane flips and ∼8% of the *trans*-to-*cis* flips. Peptide-conformation correction leads to a small improvement in the *R* factors, but more importantly surprisingly often provides a better insight into the structure–function relationship.

## Figures and Tables

**Table 1:** Selection criteria for PDB entries

Selection parameter	Criterion
Experimental method	X-ray
Resolution	3.5 or better
PDB_REDO entry	Must exist
*DSSP* entry	Must be determinable
BDB entry	Must exist
Composition	At least one chain with 25 amino acids
Flip (tt+, tc, tc+, cc+, ct or ct+)	At least one detected by *WHAT IF*

**Table 2:** Selection criteria for tetrapeptides

Selection parameter for residues	Criterion
Position in structure	Not a C-terminus or an N-terminus; not adjacent to a chain break
Amino-acid type	Must be canonical
Angles, dihedrals, improper dihedrals	Must be determinable
Atoms	All must be present; all *B* factors > 0^2^; all occupancies = 1.0
Covalently bound atoms	Only canonical bonds and no other bonds, not even disulfide bonds
Anything outside own molecule	Not within 2.5 of O atom in central peptide plane

**Table 3:** Peptide-conformation data For each peptide class three rows are given. The first row shows the counting statistics for peptide-conformation differences between PDB_REDO and PDB in the 16688 structure pairs that share at least one *trans* *cis* difference or a peptide-plane flip. The flip types are illustrated in Fig. 1 ▸. The second row shows the subset of these cases that has been used to train Random Forest classifiers. The tt cases were needed to teach the method what correct (*trans*) peptide planes look like. The third row shows the independent cases used to test the method. The 1088 cases in the test set of 438 structures have been validated manually and were corrected and re-refined when necessary. The test cases have been derived from PDB_REDOPDB comparison (the test cases are not included in the first row) or were otherwise detected over the course of this study. Entries in bold indicate that *WHAT_CHECK* can now validate cases that fall into this category; for the other classes insufficient data are available for proper training and testing. The tc cases for *X*-Pro were solved with a very simple, manually designed decision tree, as explained in the text.

Peptide class		tt	tt+	tc	tc+	cc	cc+	ct	ct+
*X*-Xnpg	Found	**13875524**	**24742**	176	0	8001	0	0	0
	Train	**4307**	**4131** †	176	0	0	0	0	0
	Test	**435**	**65**	122	12	6	0	21	3
*X*-Pro	Found	**696375**	0	**dt**	**88**	33236	0	0	0
	Train	**88**	0	**dt**	**88**	0	0	0	0
	Test	**90**	1	**dt**	**69**	74	0	3	13‡
*X*-Gly	Found	**1141604**	**11869**	0	0	2329	0	0	0
	Train	**1049**	**1049** §	0	0	0	0	0	0
	Test	**77**	**31**	7¶	0	0	0	4	0

† Training and testing examples were only taken from structures solved at 2.2 resolution or better.

‡ Eight occurrences in different chains of PDB entry 1k1d (Cheon *et al.*, 2002 ▸).

§ Training and testing examples were only taken from structures solved at better than 2.0 resolution.

¶ Six occurrences in different chains of PDB entry 2ef5.

**Table 4:** Test-set performance The performance on the test set is shown for the four RF classifiers. The performances of the WH method with the original threshold and with the threshold determined in the present study, respectively, are shown in parentheses. Note that the classification accuracy is sensitive to class imbalance, while the area under the receiver operating characteristic curve (AUC) and the Matthews correlation coefficient (MCC; Matthews, 1975 ▸) are not. The values for several other performance metrics and the confusion tables for the combined predictions can be found on the project’s website.

	*X*-Xnpg		*X*-Pro	*X*-Gly
	tt+	tc	tc+	tt+
AUC	0.99	0.98 (0.97/0.97)	0.94	0.98
MCC	0.91	0.89 (0.31/0.82)	0.85	0.93
Accuracy	0.98	0.96 (0.80/0.94)	0.92	0.97

**Table 5:** *X*-Xnpg test-set predictions The rows give the true class and the columns give the predicted class. RF, the method developed in this study (MCC = 0.89). WH, the method developed by Weiss Hilgenfeld (1999 ▸) with a *D*_tot_ score threshold of 143.10 (MCC = 0.31). WH, WH with a redetermined *D*_tot_ cutoff of 82.256 (MCC = 0.82).

	RF		WH		WH	
	tc	tt	tc	tt	tc	tt
tc	107	14	15	106	110	11
tt	6	412	0	418	24	394

## References

[bb1] Almassy, R. J., Janson, C. A., Kan, C. C. & Hostomska, Z. (1992). *Proc. Natl Acad. Sci. USA*, **89**, 6114–6118. 10.1073/pnas.89.13.6114 PMC49448 1631098

[bb2] Banerjee, R., Das, K., Ravishankar, R., Suguna, K., Surolia, A. & Vijayan, M. (1996). *J. Mol. Biol.* **259**, 281–296. 10.1006/jmbi.1996.0319 8656429

[bb3] Berkholz, D. S., Driggers, C. M., Shapovalov, M. V., Dunbrack, R. L. & Karplus, P. A. (2012). *Proc. Natl Acad. Sci. USA*, **109**, 449–453. 10.1073/pnas.1107115108 PMC3258596 22198840

[bb4] Berman, H., Henrick, K., Nakamura, H. & Markley, J. L. (2007). *Nucleic Acids Res.* **35**, D301–D303. 10.1093/nar/gkl971 PMC1669775 17142228

[bb5] Bourderioux, A., Lefoix, M., Gueyrard, D., Tatibouët, A., Cottaz, S., Arzt, S., Burmeister, W. P. & Rollin, P. (2005). *Org. Biomol. Chem.* **3**, 1872–1879. 10.1039/b502990b 15889170

[bb6] Breiman, L. (2001). *Mach. Learn.* **45**, 5–32.

[bb7] Breithaupt, C., Kurzbauer, R., Lilie, H., Schaller, A., Strassner, J., Huber, R., Macheroux, P. & Clausen, T. (2006). *Proc. Natl Acad. Sci. USA*, **103**, 14337–14342. 10.1073/pnas.0606603103 PMC1586121 16983071

[bb8] Brown, K., Djinovic-Carugo, K., Haltia, T., Cabrito, I., Saraste, M., Moura, J. J. G., Moura, I., Tegoni, M. & Cambillau, C. (2000). *J. Biol. Chem.* **275**, 41133–41136. 10.1074/jbc.M008617200 11024061

[bb9] Butcher, S. J., Grimes, J. M., Makeyev, E. V., Bamford, D. H. & Stuart, D. I. (2001). *Nature (London)*, **410**, 235–240. 10.1038/35065653 11242087

[bb10] Cheng, W.-C., Chen, Y.-F., Wang, H.-J., Hsu, K.-C., Lin, S.-C., Chen, T.-J., Yang, J.-M. & Wang, W.-C. (2012). *PLoS One*, **7**, e33481. 10.1371/journal.pone.0033481 PMC3306394 22438938

[bb11] Cheon, Y.-H., Kim, H.-S., Han, K.-H., Abendroth, J., Niefind, K., Schomburg, D., Wang, J. & Kim, Y. (2002). *Biochemistry*, **41**, 9410–9417. 10.1021/bi0201567 12135362

[bb12] Colby, T. D., Vanderveen, K., Strickler, M. D., Markham, G. D. & Goldstein, B. M. (1999). *Proc. Natl Acad. Sci. USA*, **96**, 3531–3536. 10.1073/pnas.96.7.3531 PMC22327 10097070

[bb13] Croll, T. I. (2015). *Acta Cryst.* D**71**, 706–709. 10.1107/S1399004715000826 25760617

[bb14] Di Costanzo, L., Ilies, M., Thorn, K. J. & Christianson, D. W. (2010). *Arch. Biochem. Biophys.* **496**, 101–108. 10.1016/j.abb.2010.02.004 PMC2850953 20153713

[bb15] Emsley, P., Lohkamp, B., Scott, W. G. & Cowtan, K. (2010). *Acta Cryst.* D**66**, 486–501. 10.1107/S0907444910007493 PMC2852313 20383002

[bb16] Engh, R. A. & Huber, R. (2001). *International Tables for Crystallography*, Vol. *F*, edited by M. G. Rossmann & E. Arnold, pp. 382–392. Dordrecht: Kluwer Academic Publishers.

[bb17] Evans, J. C., Huddler, D. P., Hilgers, M. T., Romanchuk, G., Matthews, R. G. & Ludwig, M. L. (2004). *Proc. Natl Acad. Sci. USA*, **101**, 3729–3736. 10.1073/pnas.0308082100 PMC374312 14752199

[bb18] Exarchos, K. P., Papaloukas, C., Exarchos, T. P., Troganis, A. N. & Fotiadis, D. I. (2009). *J. Biomed. Inform.* **42**, 140–149. 10.1016/j.jbi.2008.05.006 18586558

[bb19] Frömmel, C. & Preissner, R. (1990). *FEBS Lett.* **277**, 159–163. 10.1016/0014-5793(90)80833-5 2269347

[bb20] Gan, L., Petsko, G. A. & Hedstrom, L. (2002). *Biochemistry*, **41**, 13309–13317. 10.1021/bi0203785 12403633

[bb21] Gunasekaran, K., Gomathi, L., Ramakrishnan, C., Chandrasekhar, J. & Balaram, P. (1998). *J. Mol. Biol.* **284**, 1505–1516. 10.1006/jmbi.1998.2154 9878367

[bb22] Hayward, S. (2001). *Protein Sci.* **10**, 2219–2227. 10.1110/ps.23101 PMC2374056 11604529

[bb23] Hooft, R. W. W., Vriend, G., Sander, C. & Abola, E. E. (1996). *Nature (London)*, **381**, 272. 10.1038/381272a0 8692262

[bb24] Huber, S. K. & Scheidig, A. J. (2005). *FEBS Lett.* **579**, 2821–2829. 10.1016/j.febslet.2005.04.020 15907487

[bb25] Huber, R. & Steigemann, W. (1974). *FEBS Lett.* **48**, 2–4. 10.1016/0014-5793(74)80475-8 4435223

[bb26] Jabs, A., Weiss, M. S. & Hilgenfeld, R. (1999). *J. Mol. Biol.* **286**, 291–304. 10.1006/jmbi.1998.2459 9931267

[bb27] Jin, M. S., Kim, S. E., Heo, J. Y., Lee, M. E., Kim, H. M., Paik, S.-G., Lee, H. & Lee, J.-O. (2007). *Cell*, **130**, 1071–1082. 10.1016/j.cell.2007.09.008 17889651

[bb28] Joosten, R. P., Joosten, K., Cohen, S. X., Vriend, G. & Perrakis, A. (2011). *Bioinformatics*, **27**, 3392–3398. 10.1093/bioinformatics/btr590 PMC3232375 22034521

[bb29] Joosten, R. P., Joosten, K., Murshudov, G. N. & Perrakis, A. (2012). *Acta Cryst.* D**68**, 484–496. 10.1107/S0907444911054515 PMC3322608 22505269

[bb30] Joosten, R. P. *et al.* (2009). *J. Appl. Cryst.* **42**, 376–384. 10.1107/S0021889809008784 PMC3246819 22477769

[bb31] Joosten, R. P. & Vriend, G. (2007). *Science*, **317**, 195–196. 10.1126/science.317.5835.195 17626865

[bb32] Kabsch, W. & Sander, C. (1983). *Biopolymers*, **22**, 2577–2637. 10.1002/bip.360221211 6667333

[bb33] Klabunde, T., Eicken, C., Sacchettini, J. C. & Krebs, B. (1998). *Nature Struct. Mol. Biol.* **5**, 1084–1090. 10.1038/4193 9846879

[bb34] Kleywegt, G. J., Harris, M. R., Zou, J., Taylor, T. C., Wählby, A. & Jones, T. A. (2004). *Acta Cryst.* D**60**, 2240–2249. 10.1107/S0907444904013253 15572777

[bb35] Kundhavai Natchiar, S., Arockia Jeyaprakash, A., Ramya, T. N. C., Thomas, C. J., Suguna, K., Surolia, A. & Vijayan, M. (2004). *Acta Cryst.* D**60**, 211–219. 10.1107/S090744490302849X 14747696

[bb36] Liaw, A. & Wiener, M. (2002). *R. News*, **2**, 18–22.

[bb37] Lovering, A. L., Lee, S.-S., Kim, Y. W., Withers, S. G. & Strynadka, N. C. J. (2005). *J. Biol. Chem.* **280**, 2105–2115. 10.1074/jbc.M410468200 15501829

[bb38] MacArthur, M. W. & Thornton, J. M. (1991). *J. Mol. Biol.* **218**, 397–412. 10.1016/0022-2836(91)90721-h 2010917

[bb39] Matthews, B. W. (1975). *Biochim. Biophys. Acta*, **405**, 442–451. 10.1016/0005-2795(75)90109-9 1180967

[bb40] McCammon, J. A., Gelin, B. R. & Karplus, M. (1977). *Nature (London)*, **267**, 585–590. 10.1038/267585a0 301613

[bb41] McNicholas, S., Potterton, E., Wilson, K. S. & Noble, M. E. M. (2011). *Acta Cryst.* D**67**, 386–394. 10.1107/S0907444911007281 PMC3069754 21460457

[bb42] Moustafa, I., Connaris, H., Taylor, M., Zaitsev, V., Wilson, J. C., Kiefel, M. J., von Itzstein, M. & Taylor, G. (2004). *J. Biol. Chem.* **279**, 40819–40826. 10.1074/jbc.M404965200 15226294

[bb43] Murshudov, G. N., Skubák, P., Lebedev, A. A., Pannu, N. S., Steiner, R. A., Nicholls, R. A., Winn, M. D., Long, F. & Vagin, A. A. (2011). *Acta Cryst.* D**67**, 355–367. 10.1107/S0907444911001314 PMC3069751 21460454

[bb44] Pahlke, D., Leitner, D., Wiedemann, U. & Labudde, D. (2005). *Bioinformatics*, **21**, 685–686. 10.1093/bioinformatics/bti089 15509597

[bb45] Pal, D. & Chakrabarti, P. (1999). *J. Mol. Biol.* **294**, 271–288. 10.1006/jmbi.1999.3217 10556045

[bb46] Ramachandran, G. N. & Mitra, A. K. (1976). *J. Mol. Biol.* **107**, 85–92. 10.1016/s0022-2836(76)80019-8 1003461

[bb47] Ramachandran, G. N. & Sasisekharan, V. (1968). *Adv. Protein Chem.* **23**, 283–438. 10.1016/s0065-3233(08)60402-7 4882249

[bb48] R Core Team (2014). *R: A Language and Environment for Statistical Computing.* R Foundation for Statistical Computing, Vienna, Austria. http://www.r-project.org/.

[bb49] Sanz-Aparicio, J., Hermoso, J. A., Martínez-Ripoll, M., González, B., López-Camacho, C. & Polaina, J. (1998). *Proteins*, **33**, 567–576. 10.1002/(sici)1097-0134(19981201)33:4<567::aid-prot9>3.0.co;2-u 9849940

[bb50] Sanz-Aparicio, J., Hermoso, J. A., Martínez-Ripoll, M., Lequerica, J. L. & Polaina, J. (1998). *J. Mol. Biol.* **275**, 491–502. 10.1006/jmbi.1997.1467 9466926

[bb51] Schomaker, V. & Trueblood, K. N. (1968). *Acta Cryst.* B**24**, 63–76.

[bb52] Song, J., Burrage, K., Yuan, Z. & Huber, T. (2006). *BMC Bioinformatics*, **7**, 124. 10.1186/1471-2105-7-124 PMC1450308 16526956

[bb53] Stenkamp, R. E. (2005). *Acta Cryst.* D**61**, 1599–1602. 10.1107/S090744490503043X 16301793

[bb54] Stewart, D. E., Sarkar, A. & Wampler, J. E. (1990). *J. Mol. Biol.* **214**, 253–260. 10.1016/0022-2836(90)90159-J 2370664

[bb55] Stoddard, B. L. & Pietrokovski, S. (1998). *Nature Struct. Mol. Biol.* **5**, 3–5. 10.1038/nsb0198-3 9437416

[bb56] Sugiura, I., Sasaki, C., Hasegawa, T., Kohno, T., Sugio, S., Moriyama, H., Kasai, M. & Matsuzaki, T. (2004). *Acta Cryst.* D**60**, 674–679. 10.1107/S0907444904002549 15039555

[bb57] Tickle, I. J. (2012). *Acta Cryst.* D**68**, 454–467. 10.1107/S0907444911035918 PMC3322605 22505266

[bb58] Touw, W. G., Baakman, C., Black, J., te Beek, T. A. H., Krieger, E., Joosten, R. P. & Vriend, G. (2015). *Nucleic Acids Res.* **43**, D364–D368. 10.1093/nar/gku1028 PMC4383885 25352545

[bb59] Touw, W. G. & Vriend, G. (2014). *Protein Eng. Des. Sel.* **27**, 457–462. 10.1093/protein/gzu044 25336027

[bb60] Vriend, G. (1990). *J. Mol. Graph.* **8**, 52–56. 10.1016/0263-7855(90)80070-v 2268628

[bb62] Wang, M.-L., Li, W.-J., Wang, M. L. & Xu, W. B. (2004). *J. Pept. Res.* **63**, 23–28. 10.1046/j.1399-3011.2004.00100.x 14984570

[bb61] Wang, S. F., Jin, J.-Y., Zeng, Z. H. & Tian, G. R. (2010). *Chin. Chem. Lett.* **21**, 159–162.

[bb63] Wang, S.-F., Tian, G. R., Zhang, W.-Z. & Jin, J.-Y. (2009). *Bioorg. Med. Chem. Lett.* **19**, 5009–5011. 10.1016/j.bmcl.2009.07.060 19646864

[bb64] Weaver, L. H., Kwon, K., Beckett, D. & Matthews, B. W. (2001). *Proc. Natl Acad. Sci. USA*, **98**, 6045–6050. 10.1073/pnas.111128198 PMC33419 11353844

[bb65] Weiss, M. S. & Hilgenfeld, R. (1999). *Biopolymers*, **50**, 536–544. 10.1002/(SICI)1097-0282(19991015)50:5<536::AID-BIP7>3.0.CO;2-1 10479736

[bb66] Weiss, M. S., Jabs, A. & Hilgenfeld, R. (1998). *Nature Struct. Mol. Biol.* **5**, 676. 10.1038/1368 9699627

[bb67] Whitby, F. G., Luecke, H., Kuhn, P., Somoza, J. R., Huete-Perez, J. A., Phillips, J. D., Hill, C. P., Fletterick, R. J. & Wang, C. C. (1997). *Biochemistry*, **36**, 10666–10674. 10.1021/bi9708850 9271497

[bb68] Williams, L. K., Li, C., Withers, S. G. & Brayer, G. D. (2012). *J. Med. Chem.* **55**, 10177–10186. 10.1021/jm301273u 23050660

[bb69] Winn, M. D. *et al.* (2011). *Acta Cryst.* D**67**, 235–242.

[bb70] Xu, G., Cirilli, M., Huang, Y., Rich, R. L., Myszka, D. G. & Wu, H. (2001). *Nature (London)*, **410**, 494–497. 10.1038/35068604 11260720

[bb71] Yu, J. W., Jeffrey, P. D. & Shi, Y. (2009). *Proc. Natl Acad. Sci. USA*, **106**, 8169–8174. 10.1073/pnas.0812453106 PMC2688887 19416807

[bb72] Zhao, B. *et al.* (2005). *J. Biol. Chem.* **280**, 11599–11607. 10.1074/jbc.M410933200 15659395

[bb73] Zimmerman, S. S. & Scheraga, H. A. (1976). *Macromolecules*, **9**, 408–416. 10.1021/ma60051a005 940354

